# The Impact of the CFTR Gene Discovery on Cystic Fibrosis Diagnosis, Counseling, and Preventive Therapy

**DOI:** 10.3390/genes11040401

**Published:** 2020-04-08

**Authors:** Philip M. Farrell, Michael J. Rock, Mei W. Baker

**Affiliations:** 1 Departments of Pediatrics and Population Health Sciences, University of Wisconsin School of Medicine and Public Health, 600 Highland Madison, WI 53792, USA; 2 Department of Pediatrics, University of Wisconsin School of Medicine and Public Health, 600 Highland Ave, Madison, WI 53792, USA; mjrock@pediatrics.wisc.edu (M.J.R.);; 3 Newborn Screening Laboratory, Wisconsin State Laboratory of Hygiene, University of Wisconsin–Madison, 465 Henry Mall, Madison, WI 53706, USA

**Keywords:** cystic fibrosis, newborn screening, trypsinogen, *CFTR* gene, next generation sequencing, health policy

## Abstract

Discovery of the cystic fibrosis transmembrane conductance regulator (*CFTR)* gene was the long-awaited scientific advance that dramatically improved the diagnosis and treatment of cystic fibrosis (CF). The combination of a first-tier biomarker, immunoreactive trypsinogen (IRT), and, if high, DNA analysis for CF-causing variants, has enabled regions where CF is prevalent to screen neonates and achieve diagnoses within 1–2 weeks of birth when most patients are asymptomatic. In addition, IRT/DNA (*CFTR*) screening protocols simultaneously contribute important genetic data to determine genotype, prognosticate, and plan preventive therapies such as CFTR modulator selection. As the genomics era proceeds with affordable biotechnologies, the potential added value of whole genome sequencing will probably enhance personalized, precision care that can begin during infancy. Issues remain, however, about the optimal size of *CFTR* panels in genetically diverse regions and how best to deal with incidental findings. Because prospects for a primary DNA screening test are on the horizon, the debate about detecting heterozygote carriers will likely intensify, especially as we learn more about this relatively common genotype. Perhaps, at that time, concerns about CF heterozygote carrier detection will subside, and it will become recognized as beneficial. We share new perspectives on that issue in this article.

## 1. Introduction

Every physician’s first duty is to diagnose—accurately and promptly—because diagnosis is the first step of treatment. In the case of cystic fibrosis (CF), accurate and prompt diagnosis was impossible before the newborn screening (NBS) era for the majority of patients [1]. In fact, children with CF typically experienced a diagnostic odyssey [2] and suffered irreversible malnutrition [3] and/or lung disease [4] before they had their diagnoses established through sweat chloride tests. Others, perhaps 5%–10% and certainly at least 1% [5], died undiagnosed; those unfortunate patients would have had fatal hyponatremic/hypochloremic dehydration, protein-energy malnutrition, or catastrophic lung disease [3,4]. It is likely that these tragic fatalities continue in some regions of the world where CF NBS has not yet been implemented [1]. The advantages of early diagnosis are not only intuitive but were identified in clinical research as early as 1970, when Shwachman et al. [6] reported a classic study showing that survival was much better when diagnosis occurred before three months of age.

Early NBS attempts using meconium, however, failed feasibility assessment [7]. Fortunately, the use of dried blood spot screening, which was immediately successful for phenylketonuria [8], became attractive for CF when Crossley et al. [9] discovered in New Zealand that high immunoreactive trypsinogen (IRT) levels predicted a higher risk for CF. Because most regions had well-functioning systems in place for universal collection of dried blood spot specimens and their analysis in a central laboratory, the stage was set for CF NBS using IRT as an initial and, about two weeks later, a “recall” test to confirm hypertrypsinogenemia [10]. Skepticism remained, however, because of concerns about the IRT test per se, whether or not significant clinical benefits actually occurred with early diagnoses, and how much adverse impact was being imposed on parents of screened neonates, i.e., the degree of psychosocial harm [11]. In retrospect, the major issue that initially limited CF NBS acceptance concerned the IRT/IRT screening strategy (a protocol with relatively low sensitivity) using a biomarker with variable reliability [12,13]. Consequently, it became clear that more research was needed on all aspects of CF NBS and that the IRT method of screening needed to be improved. In fact, a decade after the report from New Zealand, there was still worldwide debate among health policy decision-makers whether or not CF NBS was worthwhile [1] and even doubt among organizations like the US Cystic Fibrosis Foundation (CFF)—expressed emphatically when it sponsored a negative but influential commentary [11]. Consequently, CF NBS implementation was slow in North America and Europe, and one country (France) even discontinued their national IRT-based program [1]. Suddenly, however, the situation changed dramatically, when the *CFTR* discovery was reported in September 1989 [14]. This major advance forever changed the landscape with regard to both diagnosis and treatment.

## 2. Development of the IRT/DNA Screening Strategy

*Dans les champs de l’observation le hasard ne favorise que les esprits prepares* (in the field of observation, chance favors only the prepared mind), emphasized Louis Pasteur during his lecture at the University of Lille on 7 December 1854. The story surrounding transformation from IRT/IRT to IRT/DNA in Wisconsin fulfills Pasteur’s admonition. Beginning in April 1985, after securing grants from the CFF and NIH, Wisconsin’s two CF Centers began a randomized clinical trial (RCT) of newborn screening that led to a conclusion by 1989 that the IRT/IRT test was good, but not good enough, for routine use by public health laboratories [15]. The results supporting that conclusion were in the process of being reported in *Pediatrics* at a time when the delay from manuscript acceptance (in this case, 3 July 1989) to publication was typically at least a few months. Under Conclusions, it was stated that the IRT test will not be adequate as a sole screening method but might be useful as an initial marker if followed by another tier as in the thyroxin/TSH combination for congenital hypothyroidism. However, as Rock et al. [15] awaited the page proofs, the discovery of the cystic fibrosis transmembrane conductance regulator (*CFTR*) gene and the p.Phe508del (c.1521_1523delCTT) variant was reported [14], and with the Editor’s approval, the following comment was added under a new section, entitled Speculation and Relevance: *With advances in technology and the recent identification of one of the cystic fibrosis mutations and the identification of other mutations to soon follow, we believe that the strategy for cystic fibrosis newborn screening will need to evolve into a true two-tier screening test. The first tier would be the IRT assay; if the IRT assay is positive, the second tier would be performed on the same original blood spot, and it would be a probe for the cystic fibrosis mutations. The implementation of cystic fibrosis screening, however, should be delayed until a clear benefit of newborn screening has been identified*.

While the Rock et al. [15] article was still in press, and thanks to applicable NIH grant support, Farrell recruited an excellent molecular geneticist, Ronald Gregg, and early in 1990 a reliable but laborious method was developed [16,17,18,19] to incorporate into the RCT. In summary, using a method just described by Jinks et al. [20], DNA was isolated from a 5-mm blood-soaked filter paper circle obtained from a Guthrie card, denatured, PCR-amplified, and then analyzed on polyacrylamide gel with the genotype determined according to the “rapid” method of Rommens et al. [21]. Prior to implementation of the IRT /DNA screening protocol, a pilot phase was performed to evaluate the validity of the contemplated DNA testing procedure. The Wisconsin’s team’s main concern was the possibility of error due to sample cross-contamination because of the serial sample punching procedures used by the newborn screening laboratory. Some low-level cross-contamination seemed likely to occur, because the automated punches used to prepare samples from Guthrie cards cannot be decontaminated sufficiently to destroy DNA from previously punched cards. Consequently, we were concerned that the amount of contamination might be sufficient for detection by the PCR-based assay. Blinded dried blood samples obtained previously from 300 infants with an elevated IRT, including 43 CF patients, were analyzed for p.Phe508. All the p.Phe508 homozygotes detected were known to have CF. Repeat analysis on independently collected blood samples confirmed the original genotype. Therefore, it was concluded that the very low level of cross-contamination that might occur with serial punching of cards is insufficient to interfere with the DNA analysis [17].

Next, and soon thereafter, all definitively diagnosed CF patients followed in the Madison and Milwaukee Centers were studied to determine the prevalence of p.Phe508del. Fortunately, the predominance of this principal CF-causing variant was apparent immediately. The data on 547 patients revealed that 264 (48.3%) were homozygous, 220 (40.2%) were p.Phe508del compound heterozygotes, and only 63 (11.5%) had two other CFTR variants. Thus, a two-tier screening test with IRT/DNA(p.Phe508del), using lower levels of IRT as the cutoff for the second tier, was determined to be valuable and have much better sensitivity than the 75% identified with the IRT/IRT protocol [12,15]. In addition, there are many other advantages listed in Table 1. It was particularly comforting to find that IRT/DNA eliminated the problem Rock et al. [22] had reported previously: that IRT alone identified an inordinately high percentage of premature and African American infants who were not at increased risk for CF but likely to have false positive IRT levels in initial dried blood spot testing.

**Figure 1:**
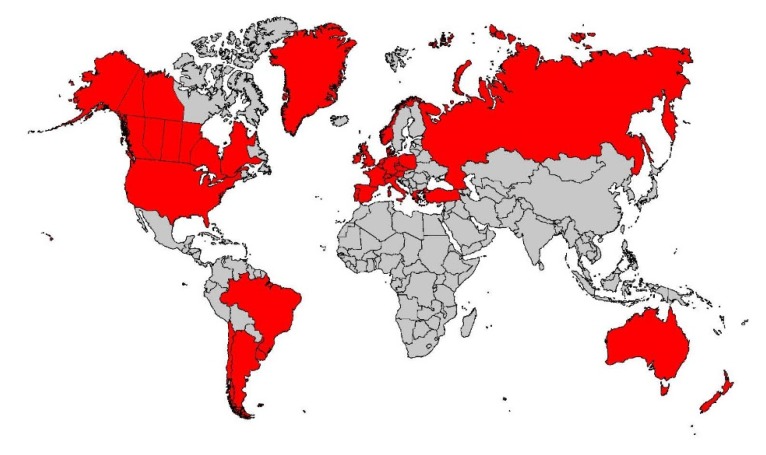
Cystic fibrosis newborn screening in 2020 around the world where the disease is prevalent.

Consequently, the Wisconsin RCT incorporated IRT/DNA as the screening test in 1991, and the project was expanded to a comprehensive epidemiologic study of childhood CF [23]. By 1991, IRT/DNA was also successfully implemented for routine screening in South Australia by Ranieri et al. [24] and soon thereafter in other regions [1], such as Brittany, where the molecular genetics expertise of Claude Férec’s laboratory was readily applied to two-tier CF NBS [25]. In retrospect, the discovery that about 90% of Europeans and Europe-derived CF populations have at least one p.Phe508del variant greatly facilitated widespread implementation of the world’s first DNA-based population screening test—the IRT/DNA(p.Phe508del) method. Its advantages and demonstration of benefits [15,26] outweighing the inevitable harms of any screening test set the stage for the Centers for Disease Control and Prevention (CDC) and the US CFF to advocate for universal CF NBS in 2004 [2] and 2005 [27], respectively. The delay in approvals by these organizations can be attributed to the fact that such advocacy was unprecedented, and both the CDC and CFF were concerned that the potential harm could outweigh the benefits [2,11,27]. These actions stimulated nationwide screening of 4 million American babies annually in less than 5 years and now worldwide screening programs, as shown in Figure 1. Moreover, a complete transformation of the care strategy for children with CF [23,28,29,30] emerged as routine early diagnosis facilitated preventive therapies, reflecting the view that prevention is far better than intervention for chronic, potentially irreversible disease. 

## 3. Improvements in IRT/DNA Screening Protocols

Although the two-tier screening test with IRT/DNA and the p.Phe508del variant only using lower cutoff levels of IRT was advantageous [12,17], and needed to be continued for the RCT, the Wisconsin team decided to change the protocol when randomization ended on 30 June 1994 after NBS-derived nutritional benefits were becoming evident [15,26]. Thus, the Wisconsin State Laboratory of Hygiene, led by Ron Laessig and Gary Hoffman, recognized that routine CF NBS should be introduced with two improvements in the screening algorithm. First, it had become clear during the RCT that the biomarker IRT was a very challenging analyte with regard to setting reliable, stable cutoff values. In fact, there were both seasonal and kit-related variations that prevented equitable use of IRT and mitigation of false negative results [12,31]. This recognition led to the floating IRT cutoff value tactic, in which the highest 4%–5% of the daily specimens were reflexed to the DNA tier [12].

In addition, recognizing the important contribution of Comeau et al. [32], many regions expanded the DNA tier to a *CFTR* multimutation panel and a sensitivity of >95% was achieved routinely (in some years, 100% sensitivity) [18]. In addition, the quality of screening improved significantly by not only allowing test completion on the initial dried blood spot specimen, thus improving timeliness, but providing valuable information on most patients’ *CFTR* variants. It was quickly learned with IRT/DNA(*CFTR*) that the vast majority of CF cases can be presumptively (genetically) diagnosed within a week of birth from the initial blood specimen and valuable genetic data obtained to predict pancreatic functional status [33]. Later, the rapid genotyping capability of IRT/DNA (*CFTR*) screening also provided guidance for selection of CFTR modulators which can now be used to achieve organ preservation if begun early [34]. On the other hand, the *CFTR* multi-mutation tactic increases the number of incidental findings, particularly detection of CF heterozygote carriers, an increasingly important topic that is discussed in detail subsequently, and identification of those with CRMS/CFSPID (cystic fibrosis transmembrane conductance regulator-related metabolic syndrome/cystic fibrosis screen positive, inconclusive diagnosis) [35,36], which is well reviewed elsewhere [37] and beyond the scope of this article.

Concerns about the higher costs of DNA/*CFTR* analyses and increased carrier detection have led to another change in regions wedded to collecting two dried blood specimens, namely the IRT/IRT/DNA protocol [38]. This algorithm modification achieves those two objectives (lower costs and fewer carriers detected) but delays diagnoses and does not improve sensitivity [39]. In view of the early risks of CF in infants such as potentially fatal hyponatremic/hypochloremic dehydration [40], and the likelihood of early malnutrition [41], it has become clear that timeliness in diagnosing the disease is an imperative. Thus, more research is needed on comparing the impact of IRT/DNA with IRT/IRT/DNA protocols. Similarly, algorithms employing pancreatitis-associated protein (PAP) and other supplemental tests need to be investigated further [1,35]. These algorithms may add potential advantages such as providing a “safety net” [35].

## 4. Application of Next Generation Sequencing

Despite its great advantages and popularity, there are many imperfections of IRT/DNA screening. One of the biggest challenges is a lower-than-ideal positive predictive value (PPV), resulting in as many as 10 heterozygote infant carriers (i.e., those with high IRT levels and single identified *CFTR* variants) for every CF case diagnosed after follow-up sweat testing. Although a 10:1 ratio may be disconcerting, other NBS tests lead to a higher proportion of false positive results such as screening for congenital adrenal hyperplasia [42]. In CF, however, the situation is exacerbated by the need for confirmatory sweat testing aa well as the occurrence of an insufficient quantity of sweat collected or an inconclusive sweat test outcome in at least 10% of the screen-positive infants. In addition, the *CFTR* panels in general use during the past two decades have had insufficient CF-causing variants to allow the detection of minority populations in many regions where uncommon CF-causing variants occur such as M1101K (c.3302T>A), found in Hutterite populations [43], and H199Y (c.595C>T) or S492F (c.1475C>T) seen in Hispanic populations [44]. As the genetic diversity of populations increases, the panels used for IRT/DNA screening will not adequately meet the goal of avoiding inequities, because missed cases are more likely among non-Caucasians. These challenges led CF NBS laboratories [45,46] to consider biotechnologies that would greatly expand the *CFTR* panel after two major advances that facilitated such a change—the advent of next generation sequencing (NGS) [47] and the knowledge gained from the CFTR2 project [48,49].

NGS, a deep, high-throughput, massive parallel DNA sequencing technology, has become popular during the past decade for genetic and genomic sequencing. Rapid progress in NGS chemistry and instrumentation coupled to the simultaneous development of advanced bioinformatics methods makes NGS feasible in public health laboratories that are responsible for NBS. Baker et al. [45] reported their successful experience with using NGS to increase the detection capability of *CFTR* pathogenic variants using DNA isolated from routine dried blood spot specimens. The NGS assay was designed to sequence all the coding regions, intron/exon boundaries, and selected intronic regions. The data analysis software was designed to mask much of the sequence data and reveal only the predetermined CF-causing variants in the *CFTR* gene as characterized by the CFTR2 project [48,49]. The significance of this study includes: (1) it was the first report from a public health NBS laboratory regarding the technical feasibility of applying NGS in routine NBS practice; and (2) the reported laboratory-developed DNA isolation method in this study is applicable to other molecular testing currently used in public health NBS laboratories, such as multiplexing real-time PCR assays to screen for severe combined immunodeficiency (SCID) [50] and spinal muscular atrophy (SMA) [51,52].

Built on the technical feasibility of applying NGS in routine NBS practice, the Wisconsin NBS program has, since April, 2016, further implemented the NGS *CFTR* variant panel as the routine second-tier testing method following first-tier IRT testing. Any newborn with one or more CF-causing variants identified is referred for a follow-up sweat chloride test as confirmatory testing. Recently, NGS data were reanalyzed on the infants with sweat chloride concentrations greater than 30 mmol/L after one CF-causing variant was identified through a regular NBS screening protocol. The NGS data reanalysis was performed by removing preset panel restrictions and viewing all variants. NGS data reanalysis is a cost-effective practice for identifying a second pathogenic or likely pathogenic *CFTR* variant in infants with a likely CF or CRMS/ CFSPID diagnosis [37]. When a single, second *CFTR* variant is detected with sequencing via NGS in a patient with a positive sweat test and/or symptoms of CF, it is quite likely to be pathogenic. Moreover, mutations in some classes like a Class I premature termination codon (PTC) can be assumed to be pathogenic; as Sosnay et al. state, “the assumption that variants predicted to introduce a PTC are deleterious is commonly accepted practice” [48]. The current *CFTR* variant panel in Wisconsin includes 328 CF-causing variants and the most common deletions (exon 2–3 deletion and exon 22–23 deletion) but not one deep intronic variant and other large deletions and duplications; this may be increased further in the future as the more CF-causing variants are added to the CFTR2 website. The State of New York also uses NGS and includes a third-tier *CFTR* sequence step before reporting all pathogenic and probable pathogenic variants prior to sweat testing, i.e., an “IRT/DNA/SEQ” algorithm [46], that markedly improves PPV.

## 5. The Potentially Added Value of Genomic Sequencing

*The not-too-distant future holds many more opportunities for discovery of CF gene modifiers … whole-genome sequencing will expand GWAS-type studies to rare variant analyses* [53]. NGS has made whole genome sequencing (WGS) feasible and affordable with platforms that are commercially available and proven reliable. Compared to whole exome sequencing, WGS is preferable, because the former only covers about 1.4% of the genome and will miss loci of potential clinical importance. Additionally, the modest cost of WGS now available is compelling. In fact, the cost of WGS is already similar to what many hospitals charge for bilateral sweat chloride tests. Clinical applications have therefore become attractive. Its use for diagnoses in critically ill newborns has already been established so that it will eventually become routine in neonatal intensive care units [54,55].

Although improvements in the diagnosis and treatment of CF have led to better outcomes for the majority of patients, the impact of pancreatic insufficiency, causing malnutrition and the occurrence of early obstructive lung disease with recurrent respiratory infections, can make identifying appropriate therapies challenging in many patients. In fact, some children labeled “non-responders” do not respond well to early GI/nutritional treatment, and they also have worse lung disease [56,57]. These observations underscore a gap in our understanding of what else will be needed to ensure successful therapeutic strategies, especially preventive care. Although the development of CFTR modulators has begun to revolutionize the treatment of CF patients, variable responses to these expensive modalities has created another important gap that needs to be addressed. With universal NBS for CF identifying presymptomatic patients, better opportunities have emerged for closing these gaps. Thus, Wisconsin recently launched a project entitled “Assessing the Added Value of Whole Genome Sequencing in Cystic Fibrosis Newborn Screening” to address the hypothesis that identifying non-*CFTR* genetic variants in individual patients could enlighten therapeutic decision-making [58]. Particular attention is being given to potential genetic modifiers of lung disease and nutrigenomic/pharmacogenomic variants related to common medications.

Exploring nutrigenomic and pharmacogenomic variants that are CF-relevant and can be readily identified with WGS appears to be fruitful according to our preliminary data [58,59]. A recently published study of siblings with different lung disease manifestations but an identical *CFTR* genotype predicting severe disease (p.Phe508del/CFTRdele2,3) generated WGS data that were informative [58]. The patients’ variable phenotypes were accompanied by a surprising degree of differences in both genetic modifier and pharmacogenomics variants. In addition, in another study designed to gain insights about the variable response of CF patients to vitamin D supplements, WGS data were obtained and analyzed in 20 children whose 25-hydroxycholecalciferol levels varied during 4–24 months of treatment. Using a polygenic score technique to assess six informative loci, Lai et al. [59] found a significant nutrigenomic correlation. This study is currently being expanded with inclusion of more children and also adults with CF. Further research will be needed and is likely to be completed in the near future to determine if genomics added to DNA-based screening/genotyping will be valuable.

## 6. Prospects for a Primary DNA Screening Test

Many regions have implemented NBS for CF using the IRT/DNA two-tier protocol with a limited panel of variants [1]. According to 14 years of Wisconsin screening data, this protocol has had approximately 97% sensitivity, and the false negatives were due mainly to low IRT levels [31]. To avoid the false negative results caused by low IRT levels, a primary DNA-based screening test could be a potential solution. From the technical perspective, DNA-based assays as primary screening tests have been done routinely in screening for SCID [50] and SMA [51,52]. For SCID, although measuring T-cell receptor excision circles (TRECs) uses molecular techniques, it does not provide specific genetic information about the individual. TRECs are small circles of DNA created in T cells during their maturation in the thymus. Their presence indicates maturation of T cells; TRECs are reduced in SCID [50]. For SMA, the screening assays are designed to assess for deletions in the survival of motor neuron 1 (SMN1) gene. Undetectable SMN1 by real-time PCR indicates a high risk for SMA due to homozygous deletion of exon 7. This condition was recently added to the US Recommended Uniform Screening Panel (RUSP) [52]. The screening test for SMA is genetic testing, but the strategy of only targeting *SMN1* absence makes it possible to avoid SMA carrier identification in newborns. Success with SMA primary DNA-based screening, however, has created interest in applying the same strategy for CF NBS when analytical biotechnology advances enough to enable fast, affordable complete *CFTR* analysis for CF-causing variants, as it inevitably will and perhaps not very far into the future. Even if/when a primary DNA screening test for CF becomes technically feasible, unavoidable CF carrier identification will continue to be a challenging situation.

## 7. Incidental Findings and Implications of Emerging Data for Genetic Counseling

An important impact of the *CFTR* gene discovery relates to CF heterozygote carriers who are now being identified in countless numbers through prenatal and neonatal screening. One of the concerns about expanding *CFTR* panels beyond the previously common 23 CF-causing variants [60] has been the increase in incidental findings, particularly heterozygous infants, i.e., false positives with high IRT levels, one *CFTR* variant, and negative sweat tests. The PPV of the IRT/DNA test ranges from about 10% to 63% depending on the algorithm and the number of CFTR variants in the panel [61]. Detecting up to 10 carriers for every CF case diagnosed has been disconcerting for some, while others consider this an added benefit of CF NBS. Those in the former camp argue that that the purpose of CF NBS is identification of affected CF patients, and some have suggested that only more severely affected children should be the target population [62]. On the other hand, CF specialists who readily tolerate, or even advocate for, carrier detection as a byproduct of NBS can point out the many parents who have regarded that outcome as beneficial and appreciated the genetic counseling. In rare instances, an older sibling with CF has also been found in association with a false positive NBS result, and in most cases better informed reproductive planning has ensued from the genetic counseling. With greatly expanded *CFTR* panels and the prospects of a primary DNA/*CFTR* screening test, the issue of detecting infants who are carriers takes on greater importance and deserves detailed consideration.

There has been relatively little research done on the CF heterozygote carrier and no previous review of the published data and their implications. In a recent international study, it was determined that the p.Phe508del variant arose in Western Europe at least 5000 years ago [63,64]. Data on the time to the most recent common ancestor also revealed that it spread from west to east during the *longue durée* of the Bronze Age [65] and into the Iron Age and beyond [64]. Hazardous environmental exposures were numerous over that period [65]. Linking the p.Phe508del/wild type individual to a selective advantage has not yet been possible, despite some efforts [66], but there can be no doubt that CF heterozygotes must have had a selective advantage [67]. The high prevalence of p.Phe508del among native Europeans implies that the p.Phe508del/wild-type individual has a heretofore undiscovered health, survival, or fertility advantage. However, when parents ask questions in false-positive cases about what CF carrier status might mean for their baby, we have lacked answers. Nevertheless, this situation seems to be changing, as some research has focused on the CF carrier.

Certainly, it has been known for many years that IRT levels in infants who prove to be carriers are higher than the general population of screened babies [68,69]. This manifestation of *CFTR* variant/wild type status was initially surprising but is now well accepted. It agrees with earlier observations that CF carriers have higher sweat chloride levels than normal [70,71]. As long ago as 1962, a study by di Sant’Agnese and Powell [70] revealed an impressive difference between 97 obligate CF carriers (parents of patients with CF) and 117 “unselected adult controls” (mean sweat chloride values of 32 and 17 mmol/L, respectively; *p* < 0.01), suggesting that the sweat electrolyte abnormality should be considered a subclinical phenotypic manifestation in the CF heterozygote. In addition, taking advantage of NBS follow up data, Farrell and Koscik [72] showed conclusively that, after controlling the age factor, CF heterozygote carriers with the p.Phe508del variant have significantly increased sweat electrolyte concentrations, although they were not high enough to be in the range diagnostic of the disease. In retrospect, this should not be surprising since as Miller et al. [73] point out “carriers have ∼50% as much CFTR anion channel activity as controls... and in some epithelia, anion transport is known to be reduced in carriers.”

With regard to symptoms and disease risk, Wang et al. [74] reported that adult obligate CF heterozygotes have a much higher prevalence of chronic rhinosinusitis than the general population. Others have reported a higher incidence of pancreatitis in genetically proven CF carrier adults [75,76]. When disorders such as chronic pancreatitis occur in these patients, they might be better classified as having a CFTR-related disorder (CFTR-RD) [77], rather than simply being labeled CF carriers, but recent observations [73,75,78] may stimulate reassessment of terminology. In addition to chronic sinusitis, three phenotypes are included in the CFTR-RD category: CBAVD (congenital bilateral absence of the vas deferens) causing male infertility, acute recurrent or chronic pancreatitis, and bronchiectasis [77]. The prevalence of these and other conditions in CF carriers is surprisingly high as discussed below.

In 2011, Tluczek et al. [78] first identified a higher risk of disease in CF carriers. Specifically, based on evaluating children with a high IRT plus one CF-causing variant and a negative sweat test, Tluzcek et al. [78] observed that these carriers have more health system encounters for documented illnesses during their first year than healthy infants with negative NBS results. More recently, Miller et al. [73] published a provocative study involving 18,902 carriers matched with controls and reported that those with one *CFTR* variant have a significantly increased risk for 57 of 59 CF-related diagnostic conditions based on odds ratio analyses. The relative risks were increased for some conditions previously associated with CF carriers (e.g., pancreatitis, male infertility, and bronchiectasis), but others were not previously suspected, such as diabetes, constipation and cholelithiasis. Although the clinical significance of their findings remains to be determined, being identified as a CF carrier may promote attention to avoiding other disease-risk factors (e.g., the importance of avoiding heavy alcohol consumption in those who are intrinsically susceptible to pancreatitis). Moreover, Miller et al. [73] pointed out that identifying CF carriers may provide rational treatment options in the future for symptomatic carriers using drugs designed to enhance CFTR function. On the other hand, when applying these results to genetic counseling, it is important to keep in mind the differences between relative and absolute risk. As Miller et al. [73] point out, the relative risk for chronic pancreatitis is high with an odds ratio of 6.76 [95% CI, 4.87–9.39] but the absolute risk is less than 1% (only 0.429 per 100 carriers). However, when all the CFTR-RD conditions [74,77] are considered in male carriers based on the data of Miller et al. [73], the cumulative absolute risk is 19.07 per 100 CF heterozygotes—not a trivial prevalence.

Thus, in addition to having a probable health benefit that explains their high frequency, CF carriers appear to be at increased risk for some diseases because of their partial CFTR dysfunction. To place this situation in perspective and learn from previous experiences, we need to consider the situation with sickle hemoglobin heterozygotes—individuals labeled as SCT for sickle cell trait. For more than a half-century, they have been identified in a variety of screening programs. After NBS for hemoglobinopathies via cellulose acetate electrophoresis became routine in the US during the late 1970s [79], numerous follow-up system problems occurred initially, particularly among African-American families. These included confusion, inappropriate labeling, stigmatization, and mistreatment. Over time, however, better follow-up communication practices have improved outcomes. Now, with data that clearly show serious health risks of SCT and benefits of early recognition, the advantages of heterozygote detection are generally accepted [80,81]. Thus, recognizing that SCT is actionable, health care systems have learned more about how to manage SCT-related communications and ensure effective, timely counseling [82]. Because the severe risks are delayed beyond childhood, when precipitating factors may supervene, such as dehydration during strenuous athletic events [80,81], counseling for SCT risks that occurs immediately after NBS must be repeated and target the heterozygous individual at an appropriate time [82]. The benefits are undeniable, however, especially the prevention of sudden death by simple hydration [80,81,82,83].

When considered collectively, the observations reviewed above suggest that we should not be describing CF carriers as completely healthy. Although providers who argue against incidental CF carrier detection do not recognize its advantages and regard these CF NBS incidental findings as “unwanted,” the benefits of counseling parents of infant carriers are clear [84,85]. As more information is learned about the CF heterozygote condition, we suggest that attitudes and practices may need to change and ensure truthful education and effective communications about the implications of CF carrier status. Although the initial genetic counseling may eventually need to include the possibility that heterozygote infants have a higher risk of CFTR-RD conditions [73,74,75,76,77], incidental detection of carrier status in false-positive infants does not yet seem actionable for the child because of the low absolute risks and thus the expectation that most CF heterozygotes will be healthy—at least until later in life. Consequently, as with SCT, the counseling will have to be timed appropriately and target the carrier when risk factor mitigation could be valuable and CFTR modulators potentially useful for severe disorders like pancreatitis [75,76].

## 8. The Opportunities for Preventive Therapies

*Management of CF disease has traditionally relied on symptom-based treatments* [86]. Thus, a “one size fits all” philosophy has been common for many years, in which a plethora of drugs are being used in standard doses. Malnutrition and growth faltering, obstructive lung disease with suppurative respiratory infections, and unresponsive pulmonary exacerbations are challenging to treat, especially in “non-responders [56,57]. Thus, preventive therapies are increasingly attractive and feasible. There are four problems in CF that are amenable to prophylactic strategies, namely: (1) salt loss in sweat that can cause fatal hyponatremic/hypochloremic dehydration; (2) pancreatic insufficiency; (3) malnutrition; and (4) chronic obstructive lung disease with recurrent infections [4]. Although excessive salt loss may seem minor and can be readily prevented with salt supplements, CF patients still die from this problem, and the breast-fed infant is especially vulnerable in hot weather. Treatment with a lifetime of daily salt supplements is effective and essential, even when a patient is taking CFTR modulators [28]. Pancreatic insufficiency and its sequelae (risk of malnutrition, recurrent pancreatitis, and diabetes mellitus) have always been considered a permanent component of CF for the majority of patients with susceptible genotypes. To most CF specialists’ surprise, however, CFTR modulator therapy has been associated with at least partial preservation of pancreatic function in recent studies examining this issue [34,84,85,86]. In fact, the weight gains of CF patients in the original trials of ivacaftor suggested its potential to reduce the detrimental impact of pancreatic insufficiency [28], and subsequent observations support this hypothesis [87,88,89]. Prevention of malnutrition in most children with CF diagnosed early in the Wisconsin RCT was achieved with an aggressive approach to nutritional management, emphasizing the combination of pancreatic enzyme replacement therapy, high caloric intake, and supplements of fat soluble vitamins plus essential fatty acid supplements [30,90]. On the other hand, some children were found to be “non-responders” to this regimen [56,57,91], and this has led to more research [59]. Lastly, the most important target for preventive therapy is the respiratory system, because the quality and quantity of life in individual patients’ experience usually depends on the severity of lung disease. Thus, the most exciting aspect of CFTR modulator therapy is its potential for ameliorating lung disease as discussed in detail elsewhere [28,88,89]. For CF patients, therefore, the transformation from intervention to prevention, associated with early diagnosis and novel treatments, is clearly the most impactful result of the discovery of the *CFTR* gene.

## 9. Conclusions

The discovery of the *CFTR* gene was the scientific advance that improved our ability to diagnose CF rapidly, genotype patients simultaneously, predict pancreatic functional status immediately, and then plan preventive care. The era of genetic/genomic medicine has brightened the outlook for all patients with CF and especially children, who are now in most cases diagnosed while still asymptomatic.

## Figures and Tables

**Table 1:** Advantages of IRT/DNA(CFTR) newborn screening compared to IRT/IRT.

1. Increased sensitivity → improved validity
2. Accelerated screening test completion by 5–7 days → 2-week diagnoses
3. Enables simultaneous determination of genotype
a. Allowing prediction of pancreatic functional status
b. Facilitating selection of CFTR modulator for preventive therapy
4. Eliminates 2-week recall specimen collection (avoid loss of infants to follow up)
5. Avoid problem of rapidly decreasing IRT as infants “age”
6. Provides presumptive (genetic) diagnosis in at least 75% of cases
7. Facilitates planning for follow-up of IRT/DNA positive infants
a. With 2 mutations, the parents’ knowledge of probable CF prior to the
sweat test facilitates immediate education and treatment
b. Facilitates rapid interpretation of intermediate sweat chloride levels
c. With 1 mutation, there is a low (~3%) residual risk or probability of CF
8. Eliminates low APGAR false IRT positive problems due to perinatal stress, particularly in premature infants with low APGAR scores
9. Reduces or eliminates the problems associated with higher IRT levels in African American babies
10. Identifies heterozygote carrier families for the genetic counseling benefit
